# Increased Risk Perception, Distress Intolerance and Health Anxiety in Stricter Lockdowns: Self-Control as a Key Protective Factor in Early Response to the COVID-19 Pandemic

**DOI:** 10.3390/ijerph19095098

**Published:** 2022-04-22

**Authors:** Christoph Lindner, Ibolya Kotta, Eszter Eniko Marschalko, Kinga Szabo, Kinga Kalcza-Janosi, Jan Retelsdorf

**Affiliations:** 1 Faculty of Education, University of Hamburg, 20146 Hamburg, Germany; jan.retelsdorf@uni-hamburg.de; 2 Leibniz Institute for Science and Mathematics Education (IPN), 24118 Kiel, Germany; 3 Faculty of Psychology and Educational Sciences, Babeş-Bolyai University, 400084 Cluj-Napoca, Romania; ibolya.kotta@ubbcluj.ro (I.K.); eszter.marschalko@ubbcluj.ro (E.E.M.); kinga.szabo@ubbcluj.ro (K.S.); kinga.kalcza-janosi@ubbcluj.ro (K.K.-J.)

**Keywords:** COVID-19, lockdown stringency, mental health, self-control, Hungary, Romania

## Abstract

Studies provide evidence that distress, (health) anxiety, and depressive symptoms were high during the first weeks of COVID-19 lockdown restrictions, decreasing over time (possibly due to individuals’ protective psychological factors). Relations between different lockdown restrictions, mental health issues, and protective factors need to be explored, since even small lockdown effects might increase the risk of future mental health issues. We merged objective lockdown stringency data with individual data (*N* = 1001) to examine differences in lockdown effects in strict lockdown (Romania) and mild lockdown (Hungary) conditions between March and May 2020 on stressors and mental health symptoms, taking protective factors into account. The stricter lockdown in Romania revealed higher levels of perceived risk of infection, distress intolerance, and COVID-19 health anxiety. Protective psychological factors were not affected by the lockdown measures. Surpassing psychological flexibility and resilient coping, self-control proved to be the most promising protective factor. It is recommended that future research merge objective data with study data to investigate the effects of different COVID-19 lockdown measures on mental health and protective factors. Policy decisions should consider lockdown-dependent consequences of mental health issues. Intervention programs are suggested to mitigate mental health issues and to strengthen peoples’ protective psychological factors.

## 1. Introduction

In an early stage of the severe acute respiratory syndrome coronavirus 2 (SARS-CoV-2) pandemic, several countries reacted with strict lockdown and quarantine policies, causing higher levels of mental health symptoms. A higher risk of increased anxiety and depressive symptoms was reported for younger, female, lower-educated, lower-earning persons, or persons living alone [1,2,3]. Prati and Mancini [4] in their meta-analysis found small average effects of lockdowns on anxiety and depression, with heterogeneous effect sizes that might be ascribable to differences in lockdowns, samples and study designs. Small mental health effects of lockdowns (0.22 ≤ *g* ≤ 0.38) were found for studies including participants from Great Britain [5,6], China [7,8,9], and the United States of America [10]. Highest effect sizes were reported for Poland (*g* = 0.64; [11]), one of many Eastern European countries where prevalence rates for anxiety (30%) and depression (27%) increased in the general population during the coronavirus disease 2019 (COVID-19) pandemic [12]. However, the findings of Zhang et al. [12] indicate heterogeneity of lockdown-dependent mental health symptoms among different populations in Eastern Europe. The authors [12] assume that differences in levels of lockdown restrictions might have differentially affected individuals’ mental health and note a lack of studies on that topic for eleven Eastern European countries including Hungary and Romania. Therefore, they call for further research gaining deeper insights into the relations between country-specific differences in COVID-19 restrictions and mental health issues. Furthermore, the role of mediating variables such as the perceived risk of a SARS-CoV-2 infection, or distress intolerance, remains unclear [4]. Initial studies show that the COVID-19 lockdowns are associated with health-related anxiety (e.g., [5]) and negative automatic thoughts (e.g., [6]) due to increased distress (intolerance) or perceived risk of getting infected. Gaining deeper insights into these relations is important since COVID-19-dependent health anxiety and negative automatic thoughts might be prerequisites for illness anxiety disorder, dysthymia, or depression [13,14,15].

During the lockdowns between March and May 2020, however, the initially high levels of anxiety and depressive symptoms decreased rapidly [1,2,3], indicating that protective psychological factors may help with adapting to the circumstances (cf. [4]). Prati and Mancini [4] call for studies investigating in more detail the interplay between different levels of lockdown measures, induced stressors, early mental health issues, and personal characteristics. Zhang et al. [12] mention that among other Eastern European countries, Hungary and Romania had not yet been subject to a single study on this topic up until today. In addition to studying differences in perceived risk of SARS-CoV-2 infection, distress intolerance, COVID-19 health anxiety, and negative automatic thoughts under different lockdown conditions, in this study we aimed to examine associations between these conditions and protective psychological factors.

To investigate such relations Salanti et al. [16] suggest merging individual data with the geographical and temporal characteristics of COVID-19 lockdowns provided in the Oxford Coronavirus Government Response Tracker (OxCGRT) dataset [17]. For our research the *Stringency Index* (SI) included in OxCGRT is most important. This daily measure contains nine governmental response indicators for each country, such as school and workplace closures, stay-at-home requirements or information campaigns (for details see [18]) and ranges from 0 (no restrictions) to 100 (maximum restrictions).

### 1.1. Strict vs. Mild Lockdown Measures in Romania vs. Hungary and Their Psychological Consequences

To investigate differences in lockdown measures, induced psychological distress, mental health symptoms, and protective factors, we followed the call from Zhang et al. [12] and studied the neighboring countries Hungary and Romania which differed in the stringency of their lockdown measures (cf. [19]).

**Figure 1:**
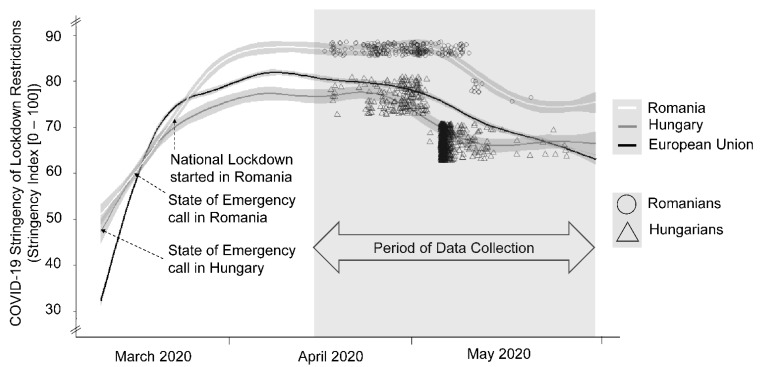
Description of the governmental stringency of lockdown conditions during the first months of the COVID-19 pandemic in Hungary and Romania. The dots and triangles represent the distribution of each individual’s SI score (not the *P*_SIW_), referring to the day of participation in the present study, and show that all participants had experienced more or less country-specific governmental restrictions for at least one month before participation.

After the state of emergency call on 11 March 2020 in Hungary [20], relatively mild personal restrictions followed until the end of May, whereas Romania (state of emergency call on 16 March 2020) introduced a strict nationwide lockdown on 24 March for the same period (cf. [21]). In Hungary small social events were permitted and people were encouraged to leave their homes only to purchase essential goods, to use medical services, for work or caregiving tasks. In Romania people were obliged to stay home and a signed statement was mandatory for people leaving their homes. The military supported the police in enforcing these restrictions (cf. [22]). The average SI between March and end of May 2020 indicates a significantly higher level of lockdown restrictions in Romania than in the European Union and Hungary (see Figure 1). It must be noted that Romania had more COVID-19 cases per million and a higher positive infection rate and reproduction rate than Hungary, whereas there were no differences in COVID-19 deaths per million and tests per thousand (details in Supplementary Materials).

To date the impact of the different lockdown measures in these countries on mental health issues is unknown [12]. Prior to the pandemic, Hungary’s prevalence rates for anxiety disorders, dysthymia, and depression in 2019 were higher than Romania’s for those under 70 and for women (IHME [23]). Following Prati and Mancini [4], even small effects of COVID-19 lockdown measures on anxiety and depressive symptoms might cause significant public mental health problems. Thus, insights into the relations between COVID-19 restrictions, induced stressors, mental health issues and personal characteristics are needed to develop mental health intervention programs (e.g., [24]).

Following Witte and Allen [25], we assume that one major psychological function of governmental restrictions is increasing people’s perception of risk of getting infected, so that they engage in preventive behaviors [26,27] such as social distancing or hand washing (e.g., [28]). In addition, lockdown-dependent perceived risk of infection might be a stressor [4] inducing depressive symptoms [29] and health anxiety [30]. Strict lockdown measures might also induce distress due to challenges such as working at home [4] or parenting [31]. Lockdown-dependent distress can exceed individuals’ distress tolerance threshold—their capacity to withstand aversive emotional distress [32]. During this pandemic, distress intolerance has been positively related to higher risks for anxiety and depression [33]. Fergus et al. [34] assume that distress intolerance is particularly associated with health anxiety.

High levels of health anxiety may lead to severe misinterpretations of bodily sensations. Regarding COVID-19, coughing for example may be interpreted as a sign of infection, in turn further increasing general anxiety [35]. Rosebrock and David [36] call for research addressing catastrophic thoughts about COVID-19 and negative automatic self-related thoughts as prerequisites for depression.

In respect of Hungary there is some evidence of higher levels of perceived risk of infection, emotional distress, and mental health symptoms during the first COVID-19 wave, between March and May 2020 [37,38], with women and younger people the most affected [39]. Comparable research in Romania is scarce. Ştefănuţ et al. [40] found Romanian females showing higher levels of depression, anxiety and distress. Mental health in Romania may have been affected more negatively than Hungary due to the stricter lockdown.

### 1.2. Protective Psychological Factors in Early Response to the COVID-19 Pandemic

To explore the possible role that psychological factors played in protecting individuals from distress and mental health symptoms during the first COVID-19 restrictions [4], we focused on three major adaptive traits (i.e., psychological flexibility, resilience and self-control) that help people dealing with environmental and social distress (e.g., cf. [41]) also during the COVID-19 pandemic.

First, psychological flexibility, the ability to adapt and to respond effectively to fluctuating situational demands by reconfiguring mental resources [42], predicted higher levels of well-being and lower COVID-19-related distress, anxiety [43,44] and problems due to social isolation [45].

Second, resilient coping, the ability to rebound from and adapt to stressors allows people to recover from or adjust to misfortune or change [46]. Resilience is associated with lower levels of depression, anxiety [47], and COVID-19-related distress [48].

Third, trait self-control—the capacity to override or change inner responses, to interrupt undesired behavioral tendencies, and to refrain from acting on them [49,50]—can play an important role during the COVID-19 pandemic. Trait self-control correlates positively with tolerance for COVID-19 generated distress [51] and with goal-directed behavior regardless of COVID-19 related uncertainties [52]. Self-control moderates the relationship between perceived severity of COVID-19 illness and mental health issues [53], is related to COVID-19 health-protective behavior and to supporting governmental regulations during lockdown [54].

Although it is assumed that all three protective psychological factors are stable traits, there is no study so far investigating whether different COVID-19 lockdown measures affected these traits. Different studies (as mentioned above) provide evidence that psychological flexibility, resilience and self-control helped people dealing with the circumstances during the first COVID-19 restrictions. However, up until today it is unknown which of these psychological factors had the strongest protective effect on mental health issues during the COVID-19 lockdowns.

### 1.3. The Present Research

First, we investigated whether stricter lockdown measures in the early COVID-19 pandemic in Romania induced higher levels of perceived risk of a SARS-CoV-2 infection, distress intolerance, COVID-19 health anxiety and negative automatic thoughts, compared to Hungary, assuming that a stricter lockdown has a stronger negative impact on these variables.

Second, we explored the relation of the different lockdown measures to protective psychological factors (psychological flexibility, resilient coping, and trait self-control), perceived risk of a SARS-CoV-2 infection, distress intolerance, COVID-19 health anxiety, and negative automatic thoughts. Particularly, we tested whether protective factors prevent negative effects on risk perception, distress intolerance, negative automatic thoughts, and COVID-19 health anxiety.

## 2. Materials and Methods

### 2.1. Participants

The sample comprised *N* = 1001 adults of the general population from Hungary and Romania with a university degree (see Table 1 for a detailed description). None of the participants had been diagnosed with COVID-19. The study protocol was approved by the Ethics Committee of the University of Babeş-Bolyai University (RO). Informed consent was obtained from all participants. Data were collected online between 15 April and 31 May 2020 using Google Forms. The study was promoted on social media (e.g., Facebook) platforms using a Snowball sampling technique. Since states of emergency were called on 11 March in Hungary and 16 March in Romania, followed by a national lockdown in Romania on 24 March 2020, participants had been confronted with governmental restrictions for at least one month before participation.

To estimate lockdown-dependent treatment effects, we applied propensity score matching (PSM), controlling for possible confounds (e.g., socio-demographics, see below). The matched sample comprised *N* = 406 (*n*_Hungary_ = 203 and *n*_Romania_ = 203) participants. We used G*Power 3.1.9.7 (Universität Düsseldorf: Psychologie-HHU, Düsseldorf, Germany) [55] for a sensitivity analysis of the paired t-tests applied, comparing outcomes between the matched samples. Given *α* = 0.05 and *N* = 406 participants, a targeted power of 0.95 would reveal small COVID-19 lockdown effects (*d_z_* = 0.179) as reported by Prati and Mancini [4] on anxiety and depression.

### 2.2. Instruments

English items were translated into Hungarian and Romanian by bilingual speakers. Cronbach’s alpha was acceptable to excellent for all used scales (0.82 ≤ *α*_Hungary_ ≤ 0.95; 0.76 ≤ *α*_Romania_ ≤ 0.94). For all measures higher scores indicate a higher level of the variable.

*Socio-demographics and COVID-19-Related Variables.* All variables (e.g., “Have you already been tested for the coronavirus?”) were assessed using single-item measures (see Table 1).

*COVID-19 Knowledge.* COVID-19 knowledge was assessed using seven items (adapted from [56]) including three correct (e.g., “The incubation period of COVID-19 can be 14 days.”) and four incorrect (e.g., “Children are most vulnerable to COVID-19.”) true-false statements.

*Official COVID-19 Dataset.* We used official governmental data provided on a daily basis between 11 March (State of Emergency call in Hungary) and 31 May 2020 in the *World in Data “Coronavirus Pandemic (COVID-19)”* dataset [17] and in the *OxCGRT* dataset [18]. We calculated personalized (weighted) stringency indices (P*_SIW_*) for the stringency conditions between 11 March and the day of participation (mean stringency index values), weighted by the derivative of the natural logarithmic function. The weight implies that P*_SIW_* scores are higher, the earlier that persons participated in the present study, since the first weeks in lockdown had the strongest negative impact on mental health issues [2]. Details about the calculation of the P*_SIW_* can be found in the statistical procedure section.

*Perceived Risk of SARS-CoV-2 Infection.* Seven items measured risk perception based on Brug et al. [57], Commodari [58] and Ibuka et al. [59]. Two items each assessed perceived likelihood of infection with SARS-CoV-2 (e.g., “I am at higher risk than others of coronavirus (COVID-19) contamination.”) and susceptibility to disease, three items the perceived severity of the disease. All items were rated on a 5-point Likert scales anchored at 1 *‘not agree at all’* and 5 *‘totally agree’*.

*Distress Intolerance.* Seven items of the frustration discomfort [60] subscale ‘emotional intolerance’ measured intolerance of emotional distress (e.g., “I can’t stand situations where I might feel upset.”) on a 5-point Likert scale ranging from 1 *‘absent’* to 5 *‘very strong’*.

*COVID-19 Health Anxiety.* The six-item brief version of the *Health Anxiety Inventory* [61] was adapted to assess COVID-19 health anxiety, including questions concerning psychological thoughts and reactions to bodily sensations (e.g., “If I hear about the new Coronavirus (COVID-19) I *never/sometimes/often/always* think I have it myself.”). Every item comprised four statements and was scored according to the choice of statements between one and four indicating level of health anxiety.

*Negative Automatic Thoughts.* The Automatic Thoughts Questionnaire [62] contains 15 statements describing negative-dysfunctional self-related automatic thoughts (e.g., “I don’t think I can go on.”). For each statement the frequencies of these thoughts were rated on a 5-point Likert scale ranging from 1 *“not at all”* to 5 *“all the time”*.

*Trait Self-Control.* Trait self-control was measured with the 13 items (e.g., “I am good at resisting temptations.”) of the *Brief Self-Control Scale* [49,50] that were rated on a 5-point Likert scale, anchored at 1 ‘*not at all like me’* and 5 ‘*very much like me’*.

*Psychological Flexibility.* Twenty items (e.g., “There are usually many possible ways to do things.”) from Ben-Itzhak et al. [63] were used to measure psychological flexibility. All items were rated on a 6-point Likert scale anchored at 1 *‘not agree at all’* and 6 *‘totally agree’*.

*Resilient Coping.* The *Brief Resilient Coping Scale* is a 4-item measure (e.g., “I actively look for ways to replace the losses I encounter in life.”) assessing resilient coping [64]. Items were rated on a 5-point Likert scale, anchored at 1 ‘*not at all like me’* and 5 ‘*very much like me’*.

### 2.3. Statistical Procedures

To examine the effects of different COVID-19 stringency conditions on our outcome variables, we combined the OxCGRT COVID-19 data [18] with the data in the present study. We calculated a weighted personalized stringency index score P*_SIW_* as follows:

(1) $$P_{SIW}\;\left(x\right)=\frac{1}{j_{\left(x\right)}}\left(\begin{array}{c} \frac{1}{n_{j\left(x\right)}}\sum_{i=1}^{n_{j\left(x\right)}}SI[y\left(x\right),\;i] \end{array}\right)\;,\;$$

where the stringency index score P*_SIW_* for person *x* is the mean value over the daily published OxCGRT stringency indices *SI* in the corresponding country *y* between 11 March 2020 (*i* = 1) and day *j,* when person *x* participated in the present study, multiplied by the weight *W. W* is $f^{\prime }\left(j_{\left(x\right)}\right)=\frac{1}{j_{\left(x\right)}}$, representing the derivative of the natural logarithmic function. We decided to use $\frac{1}{j_{\left(x\right)}}$ as a weight for the P*_SIW_* score, since Batterham et al. [1], Fancourt et al. [2] and Saunders et al. [3] reported higher levels of anxiety and depressive symptoms in the first weeks during the lockdowns in different countries, decreasing over the course of time. Fancourt et al. [2] suggest that individuals adapted to the circumstances after a few more weeks in lockdown. In line with these findings, we assumed that COVID-19-dependent personal restrictions had a more negative impact on individuals in the first weeks of the COVID-19 crisis (i.e., at the beginning of our data collection), decreasing over the course of time. The weight *W* implies that a person *x*’s P*_SIW_* score becomes higher, the earlier the person participates in the present study. Taken together, higher P*_SIW_* scores indicate on average higher levels of restrictions experienced by a person between March 11 and the day of participation, while taking the time course of the lockdown into account.

Furthermore, we applied PSM to investigate research question 1. PSM is commonly used in public health research to estimate causal treatment effects, adjusting for confounds in observational data when randomization is not possible (for an overview see [65]). In our study, we wanted to investigate marginal treatment effects [66], the propensity score being defined as the probability of treatment assignment (mild lockdown vs. strict lockdown) as predicted by all observed baseline covariates, shown in Table 1, where are also presented tests of the imbalance in baseline characteristics before PSM, using two-sample *t*-tests for continuous variables, Chi-squared tests for categorical variables, and the standardized mean differences (SMD, equivalent to Cohen’s *d*) of any covariate before and after PSM, indicating the standardized bias between both countries (cf. [67]). SMD should be reduced to a minimum, at least to |SMD| < 0.25 after PSM [68,69]. Paired *t*-tests were then applied to test differences in the outcomes after PSM [70]. We decided to include gender, age, residency, household size, number of children, employment, job status, received SARS-CoV-2 test, infected acquaintances, deaths acquaintances, COVID-19 knowledge, bank loan, self-control, psychological flexibility and resilient coping as covariates for estimating unbiased propensity scores. The selection of these covariates was mainly empirically driven, since previous research found substantial relations between them and COVID-19-related mental health symptoms [1,2,3,43,48,53,56,71]. A detailed description of how PSM was implemented in the current study is presented in Supplementary Materials.

To investigate the second research question, we applied path modeling using Mplus 8.6 (Muthén & Muthén, Los Angeles, CA, USA) [72] with the full sample (before PSM). All variables were modeled as manifest variables, and relations between all variables were allowed to vary freely. Thus, a saturated model with *df* = 0 and no fit statistics resulted. Since we were interested in indirect effects, we used the maximum likelihood estimator with bootstrapping (20,000 iterations) and confidence intervals [73]. We tested direct, indirect and total effects. Correlations between variables at the same levels (predictors, mediators, outcomes) were allowed. We included age, gender, employment, COVID-19 knowledge, received SARS-CoV-2 test, infected acquaintances and country as covariates in the model, due to substantial correlations with the outcomes (see Table 2). There were no missing data.

(All our data are available here: https://figshare.com/s/441d5df99b9120c3b6a0 (accessed on 29 December 2021)

## 3. Results

### 3.1. Governmental Restrictions and the Early COVID-19 Situation in Hungary and Romania

Referring to the World of Data COVID-19 dataset [17], the mean values of the SI for Hungary, Romania and the European Union differed significantly from zero: *F*(2, 243) = 21.250, *p* ≤ *0*.001, *η^2^_p_* = 0.146. As shown in Figure 1, the mean level of governmental restrictions was significantly higher for Romania than for Hungary (*M*_Diff_ = 8.90, *SE* = 1.44, *p* ≤ *0*.001, 95%-CI [5.58, 12.36]) and for the European Union (*M*_Diff_ = 6.84, *SE* = 1.44, *p* ≤ *0*.001, 95%-CI [3.45, 10.23]). This result indicates that, compared to the Hungarian government, the Romanian government responded more strictly to the COVID-19 outbreak.

#### 3.1.1. Research Question 1: Differences between Hungary and Romania

Before-PSM significant differences and |SMD| > 0.25 between Hungarian and Romanian participants were found in gender, age, number of children, COVID-19 knowledge, COVID-19 cases and deaths among participants’ acquaintances, self-control, psychological flexibility and resilient coping, and in the propensity score (Table 1 on the right). After PSM, SMD was reduced to a minimum (0.00 ≤ *SMD* ≤ 0.16) and the overall balance test [74] indicates optimal balancing (*χ*^2^ (15) = 5.285, *p* = 0.989) between both groups’ baseline characteristics (Table 1, on the left). Thus, differences in the outcomes are not due to these confounding variables. Detailed results of the PSM are presented in Supplementary Materials.

The significant treatment check (Table 1, at the bottom) indicates that the Romanian governmental COVID-19 restrictions between 11 March 2020 and the day of participation were stricter compared to Hungary (*d_z_ =* 1.061). The Romanian participants showed significantly higher levels of perceived risk of SARS-CoV-2 infection, distress intolerance and COVID-19 health anxiety than those from Hungary. No significant differences were found regarding negative automatic thoughts. Correlations between all COVID-19 related variables and the protective psychological factors are presented in Table 2.

#### 3.1.2. Research Question 2: Lockdown Stringency Conditions and Protective Factors as Predictors of COVID-19 Related Outcome Variables

**Figure 2:**
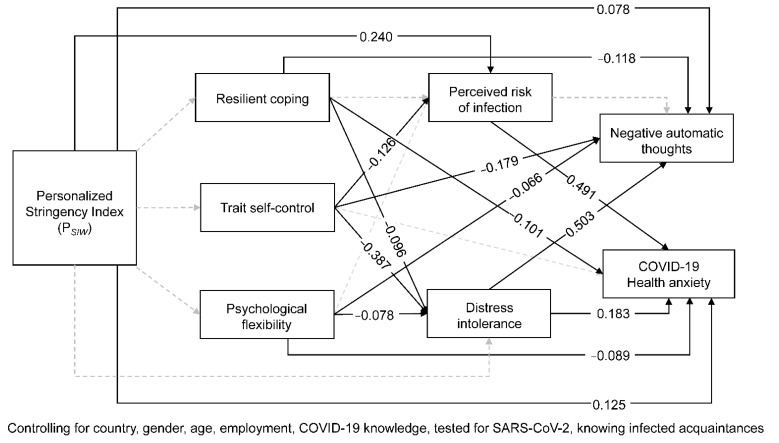
Results of the path model investigating effects of personalized stringency conditions (P*_SIW_*) and protective psychological factors on COVID-19 related outcome variables: direct effects. Correlations between variables at the same levels (predictors, mediators, outcomes) were allowed, but are omitted in favor of clarity.

The results of our path model are presented in Figure 2 and Table 3. Perceived risk of infection showed a strong relation to COVID-19 health anxiety but no significant relation to negative automatic thoughts. The latter were most strongly predicted by distress intolerance, while a weak relation was found between distress intolerance and health anxiety. The relation between P*_SIW_* and health anxiety was stronger than between P*_SIW_* and automatic thoughts. P*_SIW_* directly predicted perceived risk of infection but not distress intolerance. Perceived risk of infection mediated the relations between P*_SIW_* and health anxiety. No significant direct effect between P*_SIW_* and any of the protective psychological factors was found.

For the protective factors, weak negative relations were found between resilient coping and distress intolerance, automatic thoughts, and health anxiety. The indirect effects of resilient coping via distress intolerance to automatic thoughts and health anxiety were also significant. Higher levels of psychological flexibility were weakly and negatively related to distress intolerance, health anxiety and automatic thoughts. Also, weak, significant indirect effects of psychological flexibility on negative thoughts and health anxiety were found via distress intolerance. Resilient coping and psychological flexibility were both not significantly related to risk perception.

Trait self-control turned out to be the most promising protective factor in the early COVID-19 pandemic in Hungary and Romania, evidenced by the strongest negative direct effects on automatic thoughts, perceived risk of infection, and distress intolerance. We found significant indirect effects of self-control on health anxiety via perceived risk of infection and on automatic thoughts and health anxiety via distress intolerance

## 4. Discussion

The present study investigated psychological effects of the SARS-CoV-2 pandemic, considering different lockdown characteristics. Our first aim was to examine whether stricter lockdown measures in Romania led to higher levels of distress and mental health symptoms than did the milder restrictions in Hungary. Second, we investigated the relations between perceived risk of a SARS-CoV-2 infection, distress intolerance, health anxiety, negative automatic thoughts and the protective psychological factors psychological flexibility, resilient coping and trait self-control. To investigate whether these variables were differentially affected by contrasting lockdown measure stringency levels we followed Salanti et al. [16] in merging temporal and geographical information about lockdown restrictions with our individual data. The P*_SIW_* scores proved to be a promising measure for investigating lockdown-dependent effects. Due to the nature of lockdowns and social isolation during the pandemic, we used convenience samples, reducing the accurate representation of the respective populations in Hungary and Romania. Our sample comprised almost exclusively adult women, who were the most negatively affected by lockdown measures in the early stage of the COVID-19 pandemic [1,2,3]. Furthermore, all participants have an academic degree and >80% of them living in cities. It has to be noted that participants with severely compromised mental health also completed the survey. This was indicated by extreme values in the negative automatic thoughts questionnaire. Since we were interested in effects of different lockdown stringencies on mental health issues of the general population, participants scoring extremely high on negative automatic thoughts were excluded in all our analyses. Thus, to a certain degree all our results are representative for Romanian and Hungarian adult women with an academic degree, living in cities.

Investigating the first research question we applied PSM, controlling for personal characteristics and COVID-19 related variables as possible confounds. As hypothesized, Romanian participants living under stricter lockdown showed higher levels of risk perception, distress intolerance and COVID-19 health anxiety than did Hungarians. No significant differences were found in negative automatic thoughts. Considering that prior to COVID-19 both countries’ prevalence rates were higher for anxiety than for depression [23], lockdown-dependent effects on COVID-19 health anxiety (but not on negative automatic thoughts) might be due to a higher vulnerability to anxiety in both countries. Interestingly, prior to the pandemic prevalence rates for anxiety, dysthymia and depression were lower in Romania than Hungary, whereas we found higher levels of distress intolerance, perceived risk and health anxiety in Romania during the pandemic. Our results are in line with findings reported in a current meta-analysis [75], where stricter lockdowns were associated with higher levels of various negative emotional symptoms such as anxiety. Following the discussion by the authors, we also assume that varying levels of COVID-19-related mental health issues across countries depended on specific lockdown characteristics (e.g., length and stringency level). More precisely, the degrees of isolation, reduced social contact and general restrictions in daily life were directly caused by corresponding stringency levels of lockdown restrictions.

According to self-determination theory, satisfaction of the basic psychological needs for autonomy, social relatedness and competence is a prerequisite of well-being [76] and mental health [77]. This implies that lacking satisfaction of the basic psychological needs could be an explanation for increasing mental health issues. In stricter lockdowns, higher levels of isolation might have increased feelings of being controlled by the government, undermining the satisfaction of autonomy needs. Furthermore, we assume that reduced social contacts in stricter lockdowns led to less need satisfaction of social relatedness and reduced the possibility of gaining feedback concerning one’s own competence. Thus, lacking satisfaction of the basic psychological needs in stricter lockdowns might explain higher levels of individuals’ distress intolerance, health anxiety and other negative emotional symptoms. Since we can only speculate, further research is required investigating effects of lockdown restrictions on basic psychological need satisfaction and mental health.

Although differences in perceived risk, distress intolerance and health anxiety were small, in the long run this might result in significant public health problems (cf. [4]). Our results raise the question whether stricter lockdowns might impact people’s rational decision making and preventive behaviors. As discussed by Asmundson and Taylor [35,78] high levels of perceived risk of infection and health anxiety might lead to avoiding medical assistance, due to the risk of contagion at medical facilities. Alternatively, they might visit multiple facilities to ensure their health. Thus, rigorous controls on compliance with lockdown rules in Romania may have fostered inadequate behaviors in people with COVID-19 symptoms. Mild lockdown measures in Hungary, however, may not have led people to follow adequate preventive behaviors [27]. The different outcomes in the two countries highlight the need for well-balanced governmental lockdown strategies and effective risk communication in the context of pandemic outbreaks.

Applying path modeling to the second research question provided support for the assumptions of Witte and Allen [25]; higher levels of lockdown measures, indicated by P*_SIW_*, were related to higher levels of perceived risk of SARS-CoV-2 infection. Both variables had detrimental effects on health anxiety. As discussed above, adequate policy responses to future pandemic outbreaks should consider relations between personal restrictions, risk perception and health anxiety.

Negative automatic thoughts were most strongly related with distress intolerance, weakly with P*_SIW_* and not at all with risk perception. Stricter lockdown restrictions had no direct relation to people’s tolerance for emotional distress. This needs to be discussed in detail, since we found higher distress intolerance in Romanians than Hungarians in the PSM sample. One possible explanation may be the different statistical models used. In the path model the variance of distress intolerance was partly explainable by protective factors, which might have impeded the direct effect of P*_SIW_* on distress intolerance. Moreover, we assume that COVID-19 side effects such as worries about losing one’s job or negative attitudes toward the governmental restrictions might have negatively affected distress intolerance and mental health issues [4]. Further research may provide insights into these relations.

To the best of our knowledge, our study is the first providing empirical evidence that three key protective factors were not affected by lockdown measures. Previous research has shown psychological flexibility and resilient coping as preventive of negative automatic thoughts, health anxiety, and distress intolerance, but not risk perception [47,48,79,80].

The detrimental effects of lockdown measures were most strongly mitigated by trait self-control. Self-control was related to lower risk perception of infection. Inhibiting impulses and regulating one’s own behavior and thoughts might foster social distancing, reduce exposure to risky situations, and reduce COVID-19 health anxiety. Self-controlled individuals seem to have greater capacity for emotional tolerance, indicating that they are less prone to emotional distress, which in turn helps them regulate negative automatic thoughts and prevent health anxiety. In line with previous findings, self-controlled individuals seem to use adequate strategies to deal with COVID-19-related stressors [51], and perceived lower risk of infection may be due to higher levels of goal-orientation [52] and engagement in health-protective behavior.

Summarized, all protective factors were beneficial during the early COVID-19 pandemic. Thus, we suggest implementing public intervention programs to enhance people’s resilience, psychological flexibility, and self-control. For example, Joyce et al. [81] found that interventions combining cognitive behavioral therapy and mindfulness techniques are best to enhance resilience. Masuda and Tully [82] emphasize psychological flexibility in psychological interventions for anxiety, depressions, distress or somatization. Promoting psychological flexibility enables people to shift their focus from symptoms to underlying processes, increasing their mental health [83]. Self-control training [84] may also help. Yang et al. [85] found self-control training reduced depressive symptoms.

Against these new insights into relations between lockdown measures and psychological outcomes, we have to discuss some limitations. First, as already mentioned above our sample comprised non-COVID-19-diagnosed participants and almost exclusively adult women with an academic degree. Thus, our results may only be generalized to this group. Since various studies suggest that women in particular suffered from mental health issues during the early lockdown restrictions [1,2,3], our results may be valid for the most relevant population. Nevertheless, further studies are required to extend our research to a more demographically representative sample.

Second, PSM always neglects unobserved covariates [66]. Here, negative attitudes toward lockdown measures that may have negative effects on mental health might have been of particular interest [4]. However, during the early COVID-19 pandemic public approval of lockdown measures was high across countries [86]. Moreover, differences in cultures and economies may affect the results. Nevertheless, considering lower pre-pandemic prevalence rates for anxiety and depression in Romania than in Hungary [23], Romania’s higher levels of risk perception, health anxiety, and distress intolerance may have been affected by stricter lockdown measures.

Third, missing information about participants’ previous mental health diagnoses prevents ruling out whether the country differences are solely a function of earlier mental health issues. Nor do we know whether mental health symptoms increased during the lockdown compared to the baseline before COVID-19. Future research drawing on longitudinal studies can bring more insights into the effects of different lockdown strategies. We have tried to compensate for these weaknesses by controlling for confounds in all of our analyses, to provide initial results on relations between lockdown measures, psychological outcomes and protectors.

## 5. Conclusions

To the best of our knowledge, this is one of the first studies merging objective stringency index data with individual data to investigate effects of different lockdown measures on stressors, mental health symptoms, and protective psychological factors in the early COVID-19 pandemic. In their systematic review Zhang et al. [12] mentioned that Romania and Hungary had not been subject to a single study on that topic. Therefore, we compared these two neighboring countries with very different lockdown measures. Whereas Romania had one of the strictest lockdowns in the European Union, Hungary’s government was much less so. We found that stricter lockdown measures increased people’s perceived risk of SARS-CoV-2 infection, distress intolerance and health anxiety, while the protective psychological factors of psychological flexibility, resilient coping and self-control were not affected during the first lockdown in 2020. Furthermore, our study gained deeper insights into the relations between restriction levels, protective factors, stressors, health anxiety and negative automatic thoughts.

Policy decisions about the severity of lockdowns in pandemics should take two major issues into account. First, strict lockdowns might increase perceived risk of infection and health anxiety in the population. This might affect people’s behavior respective to medical support. On the one hand, medical facilities may be visited too little. Being infected with SARS-CoV-2 but not going to the doctor increases the risk of infecting others. On the other hand, multiple doctors might be consulted, increasing strain on health care resources. Second, mild lockdowns may lead to the population’s perception of risk being low, resulting in inadequate COVID-19 health-protective behavior. Thus, policy decisions about the stringency of lockdown measures need to consider the tradeoff between infections, deaths and healthcare capacities but also inductions on the population’s experiences and behavior. Finally, our results suggest the need to implement intervention programs to mitigate negative effects of COVID-19 lockdowns on mental health, and to strengthen protective psychological factors.

Considering specific COVID-19 pandemic characteristics and the stringency of containment policies to investigate lockdown measure effects on changes in mental health (see also [16]) is a viable option for future research. This approach might especially be useful for gaining deeper insights into the effects of different lockdown lengths and stringencies on the increased rates of unemployment in various countries over the course of the COVID-19 pandemic (e.g., [86,87]). It has to be noted that unemployment is positively related to poor (mental) health (e.g., [88,89]). Therefore, stricter and longer lockdowns might have directly affected both, mental health symptoms due to isolation, and unemployment due to business closure [87]. In addition, different lockdown characteristics could have indirectly caused detrimental effects on mental health. This would be the case if stricter lockdowns lead to a higher rate of business closure in the first place and as a consequence, to increasing prevalence rates for mental diseases due to unemployment.

## Figures and Tables

**Table 1:** Sample characteristics, baseline measures and standardized mean differences before (right; *N* = 1001) and after (left; *N* = 406) propensity score matching, and treatment effects on COVID-19 dependent variables (bottom).

		Sample *N* = 406 after PSM				Sample *N* = 1001 before PSM			
Characteristics	Category	Hungary (*n* = 203)	Romania (*n* = 203)			Hungary (*n* = 761)	Romania (*n* = 240)		
		*F_n_ (%); M (SD)*	*F_n_ (%); M (SD)*	*SMD*		*F_n_ (%); M (SD)*	*F_n_ (%); M (SD)*	*SMD*	*p*
Gender	Male	21 (10.3%)	21 (10.3%)	0.00		54 (7.1%)	31 (12.9%)	0.20	0.020
	Female	182 (89.7%)	182 (89.7%)	0.00		707 (92.9%)	209 (87.1%)	−0.20	
Age [range]		41.41 (10.02) (23–67)	40.44 (12.16) (19–79)	−0.09		50.43 (11.01) (23–81)	38.35 (12.46) (19–79)	−1.03	<0.001
Residency	Village City	27 (13.3%) 176 (86.7%)	34 (16.7%) 169 (83.3%)	0.10 −0.10		151 (19.8%) 610 (80.2%)	39 (16.3%) 201 (83.8%)	−0.08 0.10	0.251
	Household size	2.78 (1.29)	2.74 (1.10)	−0.03		2.61 (1.29)	2.71 (1.10)	0.08	0.166
	Nr. of children	1.01 (1.09)	0.92 (1.03)	−0.08		1.60 (1.21)	0.79 (1.00)	−0.73	<0.001
Education	University degree	203 (100%)	203 (100%)	0.00		761 (100%)	240 (100%)	0.00	1.000
Occupation	Unemployed	27 (13.3%)	29 (14.3%)	0.03		154 (20.2%)	32 (13.3%)	−0.18	0.102
	Employee	119 (58.6%)	119 (58.6%)	0.00		407 (53.5%)	144 (60.0%)	0.12	
	Manager	50 (24.6%)	44 (21.7%)	−0.07		163 (21.4%)	51 (21.3%)	0.00	
	CEO	7 (3.4%)	11 (5.4%)	0.10		37 (4.9%)	13 (5.4%)	0.02	
Finance	Bank loan	57 (28.1%)	62 (30.5%)	0.05		236 (31.0%)	66 (27.5%)	−0.07	0.301
COVID−19	COVID-Knowledge (Max. = 7 points)	6.11 (0.87)	6.24 (0.73)	0.16		6.13 (0.83)	6.27 (0.72)	0.18	0.022
	Already tested	2 (1.0%)	6 (3.0%)	0.14		31 (4.1%)	6 (2.5%)	−0.08	0.989
	Diagnosed	0 (0.0%)	0 (0.0%)	0.00		0 (0.0%)	0 (0.0%)	0.00	1.000
	Cases acquaintances	41 (20.2%)	43 (21.2%)	0.02		99 (13.0%)	58 (24.2%)	0.29	<0.001
	Deaths acquaintances	5 (2.5%)	5 (2.5%)	0.00		13 (1.7%)	8 (3.3%)	0.10	0.038
Protective factors	Trait self-control	3.50 (0.60)	3.52 (0.60)	0.03		3.72 (0.62)	3.46 (0.62)	−0.42	<0.001
	Psych. flexibility	4.90 (0.63)	4.88 (0.65)	−0.03		4.96 (0.65)	4.85 (0.66)	−0.17	0.033
	Resilient coping	3.83 (0.64)	3.82 (0.74)	−0.01		3.99 (0.66)	3.76 (0.76)	−0.32	<0.001
Propensity score (logit)		0.35 (0.19)	0.36 (0.20)	0.05		0.18 (0.16)	0.42 (0.23)	1.21	<0.001
		Hungary (*n* = 203)	Romania (*n* = 203)						
		*M (SD)*	*M (SD)*	*t*	*p*	*Cohen’s d_z_*			
Treatment check	P*_SIW_*	1.32 (0.14)	1.57 (0.18)	−14.975	<0.001	1.061			
				*t*	*p*	*Cohen’s d_z_*			
Dependent variables	Per. risk of infection	3.03 (0.87)	3.22 (0.88)	−2.052	0.042	0.148			
	Distress intolerance	2.41 (1.00)	2.64 (0.99)	−2.564	0.011	0.180			
	COVID Health anxiety	1.74 (0.48)	1.87 (0.44)	−2.810	0.005	0.204			
	Neg. automatic thoughts	1.79 (0.79)	1.90 (0.80)	−1.357	0.117	0.095			

*Note.* SMD, standardized mean differences before and after PSM. P*_SIW_*, personalized stringency index score weighted.

**Table 2:** Bivariate correlations for study variables.

	Variable	*M*	*SD*	01	02	03	04	05	06	07	08	09	10	11	12	13
(01)	COVID-19 knowledge	6.17	0.80	―												
(02)	Already tested (1 = yes)	0.02	0.14	0.08	―											
(03)	Cases acquaintances (1 = yes)	0.21	0.41	0.08	**0.10**	―										
(04)	Deaths acquaintances (1 = yes)	0.03	0.16	0.03	−0.02	**0.19**	―									
(05)	Perceived risk of infection	3.13	0.88	**0.22**	**0.11**	**0.14**	0.02	―								
(06)	Distress intolerance	2.52	1.00	0.03	0.08	0.02	0.04	**0.15**	―							
(07)	COVID-19 health anxiety	1.81	0.47	**0.18**	0.08	**0.10**	0.03	**0.58**	**0.29**	―						
(08)	Negative automatic thoughts	1.84	0.79	0.03	0.05	0.00	0.00	**0.19**	**0.64**	**0.29**	―					
(09)	Trait self-control	3.51	0.60	−0.08	0.00	0.07	0.08	**−0.15**	**−0.37**	**−0.14**	**−0.42**	―				
(10)	Psychological flexibility	4.89	0.64	−0.02	0.00	0.06	−0.02	0.02	**−0.26**	**−0.18**	**−0.32**	**0.19**	―			
(11)	Resilient coping	3.83	0.70	0.01	0.02	0.01	−0.07	0.03	**−0.25**	**−0.16**	**−0.38**	**0.34**	**0.60**	―		
(12)	Pers. stringency index (P*_SIW_*)	1.45	0.20	**0.13**	0.04	−0.02	0.04	**0.22**	0.09	**0.25**	**0.12**	0.01	−0.03	−0.07	―	
(13)	Age	40.93	11.14	−0.09	−0.08	**0.13**	0.06	−0.08	−0.09	**−0.16**	**−0.10**	−0.01	**0.11**	0.01	**−0.11**	―
(14)	Gender (1 = female)	0.90	0.31	−0.01	0.05	0.07	0.00	0.05	0.09	0.06	0.08	−0.01	0.06	−0.03	−0.01	−0.01

*Note.* Bold indicates *p* < 0.05.

**Table 3:** Direct, indirect, and total effects of the personalized stringency index (P*_SIW_*) and psychological protective factors on COVID-19-related outcomes.

Outcome	Direct	Indirect											Total
		via RC	via RC & PROI	via RC & DI	via TSC	via TSC & PROI	via TSC & DI	via PF	via PF & PROI	via PF & DI	via PROI	via DI	
	Personalized stringency index weighted (P*_SIW_*) to outcome												
RC	−0.086	–	–	–	–	–	–	–	–	–	–	–	−0.086
	[−0.175, 0.002]												[−0.175, 0.002]
TSC	−0.036	–	–	–	–	–	–	–	–	–	–	–	−0.036
	[−0.121, 0.049]												[−0.121, 0.049]
PF	−0.012	–	–	–	–	–	–	–	–	–	–	–	−0.012
	[−0.093, 0.067]												[−0.093, 0.067]
PROI	0.240 ***	−0.006	–	–	0.005	–	–	0.001	–	–	–	–	0.239 ***
	[0.169, 0.312]	[−0.022, 0.000]			[−0.006, 0.018]			[−0.003, 0.009]					[0.167, 0.311]
DI	−0.024	0.008	–	–	0.014	–	–	0.001	–	–	–	–	−0.001
	[−0.096, 0.047]	[0.000, 0.024]			[−0.019, 0.047]			[−0.005, 0.010]					[−0.088, 0.086]
NAT	0.078 *	0.010	0.000	0.004	0.006	0.000	0.007	0.001	0.000	0.000	0.011	−0.012	0.106 *
	[0.015, 0.142]	[0.001, 0.027]	[−0.002, 0.000]	[0.000, 0.012]	[−0.009, 0.023]	[0.000, 0.001]	[−0.010, 0.024]	[−0.004, 0.008]	[0.000, 0.001]	[−0.003, 0.005]	[0.000, 0.025]	[−0.048, 0.024]	[0.017, 0.192]
CHA	0.125 ***	0.009	−0.003	0.002	−0.002	0.002	0.003	0.001	0.000	0.000	0.118 ***	−0.004	0.250 ***
	[0.061, 0.189]	[0.001, 0.023]	[−0.011, 0.000]	[0.000, 0.005]	[−0.011, 0.002]	[−0.003, 0.009]	[−0.003, 0.009]	[−0.006, 0.010]	[−0.003, 0.009]	[−0.001, 0.002]	[0.082, 0.156]	[−0.018, 0.009]	[0.177, 0.323]
	Resilient coping to outcome												
PROI	0.074	–	–	–	–	–	–	–	–	–	–	–	0.074
	[−0.010, 0.158]												[−0.010, 0.158]
DI	−0.096 **	–	–	–	–	–	–	–	–	–	–	–	−0.096 **
	[−0.171, −0.018]												[−0.171, −0.018]
NAT	−0.118 ***	–	–	–	–	–	–	–	–	–	0.003	−0.048 *	−0.163 ***
	[−0.180, −0.055]										[0.000, 0.012]	[−0.088, −0.010]	[−0.238, −0.086]
CHA	−0.101 **	–	–	–	–	–	–	–	–	–	0.036	−0.018 *	−0.083
	[−0.171, −0.032]										[−0.005, 0.078]	[−0.035, −0.004]	[−0.172, 0.005]
	Trait self-control to outcome												
PROI	−0.126 ***	–	–	–	–	–	–	–	–	–	–	–	−0.126 ***
	[−0.192, −0.061]												[−0.192, −0.061]
DI	−0.387 ***	–	–	–	–	–	–	–	–	–	–	–	−0.387 ***
	[−0.445, −0.326]												[−0.445, −0.326]
NAT	−0.179 ***	–	–	–	–	–	–	–	–	–	−0.006	−0.194 ***	−0.379 ***
	[−0.235, −0.123]										[−0.014, 0.000]	[−0.232, −0.161]	[−0.437, −0.320]
CHA	0.059	–	–	–	–	–	–	–	–	–	−0.062 ***	−0.071 ***	−0.074 *
	[−0.001, 0.116]										[−0.096, −0.030]	[−0.098, −0.046]	[−0.141, −0.007]
	Psychological flexibility to outcome												
PROI	−0.048	–	–	–	–	–	–	–	–	–	–	–	−0.048
	[−0.124, 0.029]												[−0.124, 0.029]
DI	−0.078 *	–	–	–	–	–	–	–	–	–	–	–	−0.078 *
	[−0.145, −0.011]												[−0.145, −0.011]
NAT	−0.066 *	–	–	–	–	–	–	–	–	–	−0.002	−0.039 *	−0.107 **
	[−0.122, −0.010]										[−0.009, 0.001]	[−0.074, −0.006]	[−0.178, −0.039]
CHA	−0.089 **	–	–	–	–	–	–	–	–	–	−0.023	−0.014 *	−0.126 **
	[−0.151, −0.026]										[−0.060, 0.014]	[−0.029, −0.003]	[−0.201, −0.050]

Note. *** p < *0*.001, ** p < 0.01, * p < 0.05. RC, resilient coping; TSC, trait self-control; PF, psychological flexibility; PROI, perceived risk of infection; DI, distress intolerance; NAT, negative automatic thoughts; CHA, COVID-19 health anxiety.

## Data Availability

All data are available: https://figshare.com/s/441d5df99b9120c3b6a0 (accessed on 29 December 2021).

## Supplementary Materials

The following supporting information can be downloaded at: https://www.mdpi.com/article/10.3390/ijerph19095098/s1, Table S1: COVID-19 Characteristics in Hungary and Romania between 11 March and 31 May 2020; Table S2: Sample Sizes of the Discarded, Unmatched and Matched Participants in the Treatment and Control Conditions; Figure S1: Distributions, densities and balancing of the propensity scores before and after matching. Refs [17,65,67,68,69,70,90,91,92,93] are cited in the Supplementary file.
