# Supplementary material for: Increased Risk Perception, Distress Intolerance and Health Anxiety in Stricter Lockdowns: Self-Control as a Key Protective Factor in Early Response to the COVID-19 Pandemic
Source: Int J Environ Res Public Health. 2022 Apr 22;19(9):5098. doi: 10.3390/ijerph19095098 (PMC9100473; doi:10.3390/ijerph19095098)
Supplement: Supplementary file 1 [file ijerph-19-05098-s001.zip › ijerph-1682741-supplementary.pdf]

Supplementary Materials

Increased Risk Perception, Distress Intolerance and Health Anxiety in Stricter Lockdowns: Self-Control as a Key Protective Factor in Early Response to the COVID-19 Pandemic

Journal: International Journal of Environmental Research and Public Health

Christoph Lindner[1,2]*, Ibolya Kotta[3], Eszter Eniko Marschalko[3], Kinga Szabó[3], Kinga Kalcza-Janosi[3], &

Jan Retelsdorf[1]

[1]*University of Hamburg, Hamburg, Germany*

[2]*Leibniz Institute for Science and Mathematics Education (IPN), Germany*

[3]*Babeş-Bolyai University, Cluj-Napoca, Romania*

Supplementary Material: COVID-19 Situation in Hungary and Romania

To investigate COVID-19-dependent differences between Hungary and Romania, we used official governmental data on a daily basis between 11 March (State of Emergency call in Hungary) and 31 May 2020, as provided by the *World in Data "Coronavirus Pandemic (COVID-19)"* dataset [17]. For each variable in Table S1 we calculated mean values over the daily published data. It has to be noted that the data for Romania are available for 74 days and for Hungary for 71 days between 11 March and 31 May 2020. The one-way MANOVA in Table S1 differed to a statistically significant extent from zero, $F(6, 138) = 110.860$, $p < 0.001$, partial $\eta^2 = 0.828$, Wilk's $\Lambda = 0.172$.

Table S1. COVID-19 Characteristics in Hungary and Romania between 11 March and 31 May 2020.

	Hungary (n = 71 days)	Romania (n = 74 days)			
COVID World Data	*M (SD)*	*M (SD)*	η^2_P	F (6,138)	p
	(N = 71)	(N = 74)			
New cases/million (smoothed)	5.47 (2.57)	13.09 (4.87)	0.490	137.18	0.000
New deaths/million (smoothed)	0.74 (0.42)	0.87 (0.40)	0.024	3.50	0.063
New tests/thousand (smoothed)	0.25 (0.12)	0.28 (0.16)	0.014	1.97	0.163
Positive rate	0.03 (0.02)	0.06 (0.04)	0.275	54.17	0.000
Tests per case	59.11 (45.02)	23.60 (14.73)	0.225	41.44	0.000
Reproduction rate	1.08 (0.35)	1.22 (0.46)	0.028	4.15	0.043

Note. Cases, tests and deaths were 7-days smoothed.

Supplementary Material: Propensity Score Matching

To investigate the effects of different governmental stringency treatments on risk perception, emotional intolerance, health anxiety and negative automatic thoughts between participants from Hungary (i.e., moderate governmental COVID-19 restrictions) and Romania (i.e., high governmental COVID-19 restrictions), we applied propensity score matching (PSM) to estimate causal effects with non-experimental data [90]. Due to heterogeneous baseline characteristics and the unbalanced number of participants assigned to the treatment groups (nHungary = 761, nRomania = 240) the propensity score provides a balanced score for the treatment conditions as conditioning on the propensity score, the treatment groups are comparable in terms of the baseline covariates [65]. In our study, the propensity score is defined as the probability of treatment assignment (moderate restrictions vs. high restrictions) of condition to all observed baseline characteristics (i.e., socio-demographics, COVID-19 variables and protective psychological factors, as shown in Figure S1 on the bottom right). The estimation of each individual's propensity score is based on logistic regression analysis, where treatment assignment (Hungary vs. Romania) is the outcome variable and baseline characteristics are used as predictors. When applying PSM, ideally the correlation between treatment assignment and the covariates is removed and, therefore, treatment effects can be estimated (for extensive descriptions, see [67]). For our purposes, we examined the average treatment effects that would be seen if all or no individuals in both groups received the treatment (i.e., stricter governmental COVID-19 restrictions). Therefore, we followed Harder et al. [91] and applied the nearest neighbor matching method [92] without replacement by using subclassification (six groups). To obtain optimal matches, we set the maximum allowed difference between two participants' propensity scores (i.e., caliper) to C = 0.20 of the standard deviation of the logit of the propensity score, as proposed by Austin [70]. Following Thoemmes [94], to improve the balance on covariates, observed units outside the region of the distribution of estimated propensity scores (i.e., the area of common support) were discarded from matching in both groups. All PSM analyses were performed in IBM SPSS 22 using the psmatching tool [94]. Assessing balance in baseline characteristics before PSM we followed Austin [70] in using two-sample t-tests for continuous variables and Chi-squared tests for categorical variables to investigate group differences. Also, as recommended by Austin [67] we calculated standardized mean differences (SMD, equivalent to Cohen's d) on any covariate between the treatment and control groups before and after PSM. SMD indicates the standardized bias between both groups' baseline characteristics and should be reduced to a minimum, at least $|SMD| < 0.25$ (as recommended by [68,69]) after PSM. Unlike statistical tests of hypothesis, the SMD is not influenced by sample size, and therefore it is the optimal indicator of matching quality [67].

Table S2. Sample Sizes of the Discarded, Unmatched and Matched Participants in the Treatment and Control Conditions.

	**Sample sizes (*n*)**	
	**Hungary (Control)**	**Romania (Treated)**
All participants	761	240
Matched participants	203	203
Unmatched participants	512	28
Discarded participants	46	9

Forty-six Hungarians and 9 Romanians were outside of the area of common support. Therefore, they were excluded from matching. Unmatched participants comprised 28 Romanians and 512 Hungarians, leaving 203 participants for each of the treatment and control groups. For visual display the distributions and densities of the propensity scores before and after matching are shown in Figure S1, on the top and in the middle. On the bottom of Figure S1 the densities of the standardized mean differences before and after matching are presented. The balancing of the propensity scores for each covariate used as a predictor in the logistic regression (to estimate the propensity score) is shown on the bottom right.

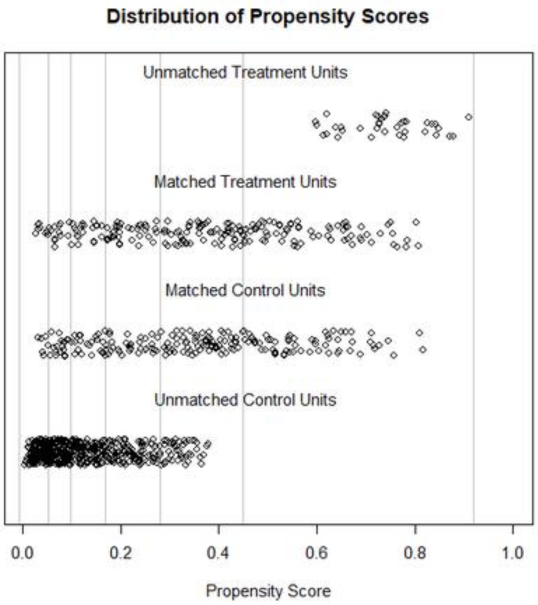

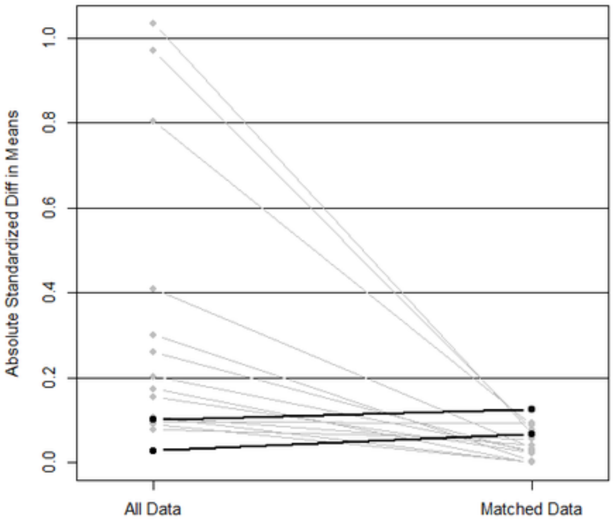

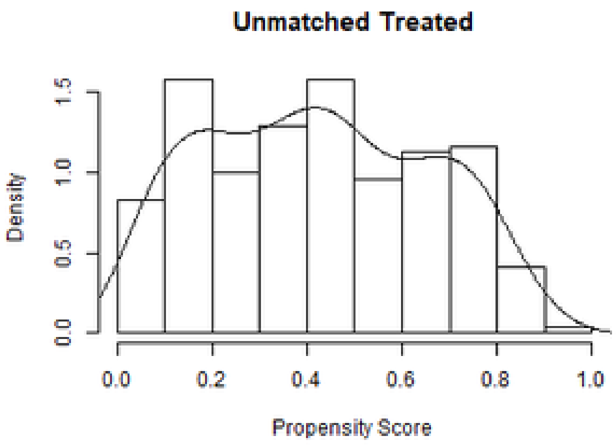
Unmatched Treated
Density
0.0 0.5 1.0 1.5
0.0 0.2 0.4 0.6 0.8 1.0
Propensity Score

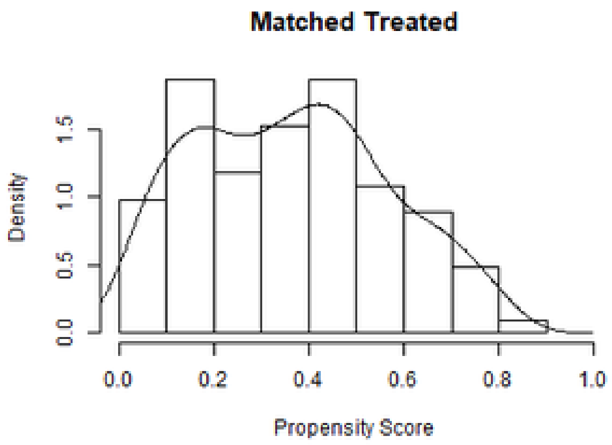
Matched Treated
Density
0.0 0.5 1.0 1.5
0.0 0.2 0.4 0.6 0.8 1.0
Propensity Score

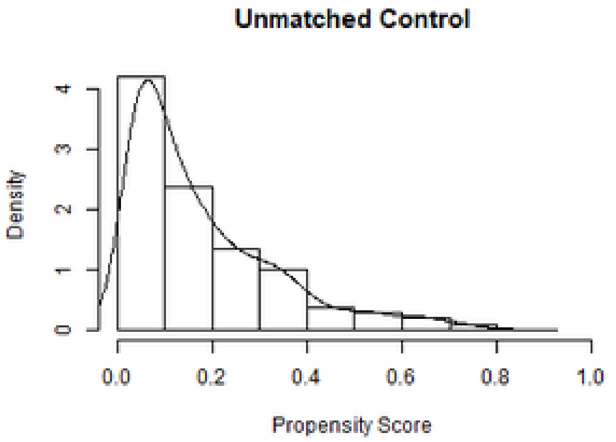
Unmatched Control
Density
0 1 2 3 4
0.0 0.2 0.4 0.6 0.8 1.0
Propensity Score

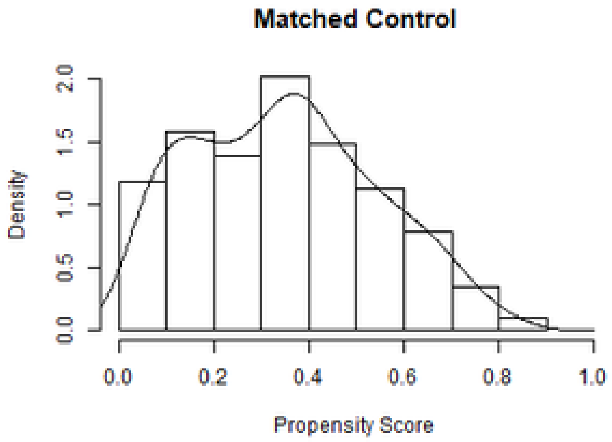
Matched Control
Density
0.0 0.5 1.0 1.5 2.0
0.0 0.2 0.4 0.6 0.8 1.0
Propensity Score

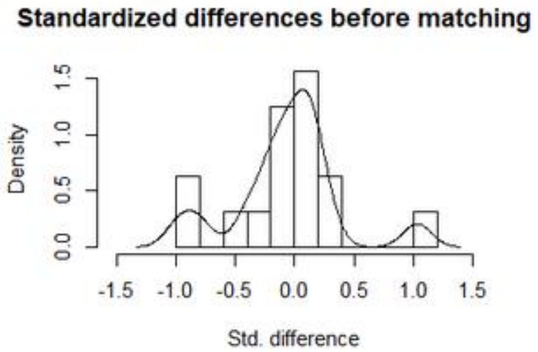
Standardized differences before matching
Density
0.0 0.5 1.0 1.5
-1.5 -1.0 -0.5 0.0 0.5 1.0 1.5
Std. difference

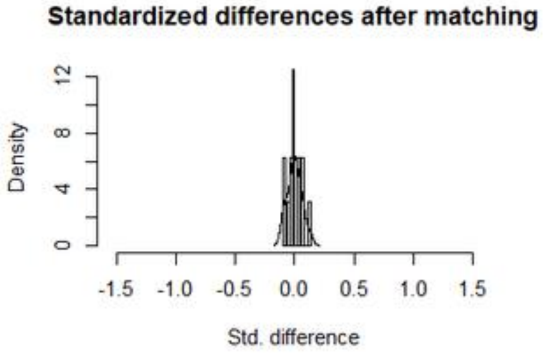
Standardized differences after matching
Density
0 4 8 12
-1.5 -1.0 -0.5 0.0 0.5 1.0 1.5
Std. difference

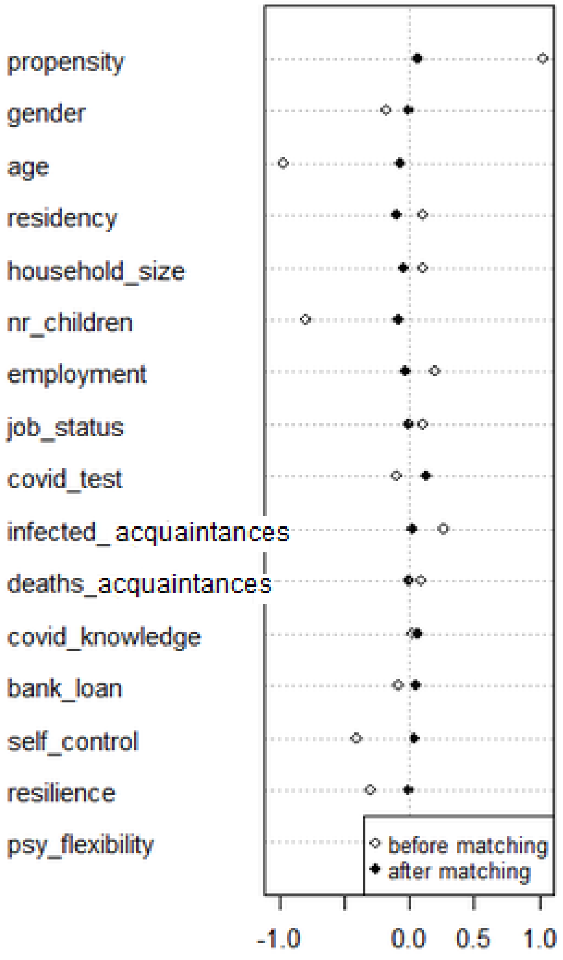

Figure S1. Distributions, densities and balancing of the propensity scores before and after matching.
